# Role of Wearable Sensing Technology to Manage Long COVID

**DOI:** 10.3390/bios13010062

**Published:** 2022-12-31

**Authors:** Kamil Reza Khondakar, Ajeet Kaushik

**Affiliations:** 1 School of Health Sciences and Technology, University of Petroleum and Energy Studies, Dehradun 248007, Uttarakhand, India; 2 NanoBioTech Laboratory, Department of Environmental Engineering, Florida Polytechnic University, Lakeland, FL 33805-8531, USA; 3 Department of Chemical Engineering, University of Johannesburg, Johannesburg 2094, South Africa

**Keywords:** long COVID, wearables, smart devices, biometrics, health profiling and management

## Abstract

Long COVID consequences have changed the perception towards disease management, and it is moving towards personal healthcare monitoring. In this regard, wearable devices have revolutionized the personal healthcare sector to track and monitor physiological parameters of the human body continuously. This would be largely beneficial for early detection (asymptomatic and pre-symptomatic cases of COVID-19), live patient conditions, and long COVID monitoring (COVID recovered patients and healthy individuals) for better COVID-19 management. There are multitude of wearable devices that can observe various human body parameters for remotely monitoring patients and self-monitoring mode for individuals. Smart watches, smart tattoos, rings, smart facemasks, nano-patches, etc., have emerged as the monitoring devices for key physiological parameters, such as body temperature, respiration rate, heart rate, oxygen level, etc. This review includes long COVID challenges for frequent monitoring of biometrics and its possible solution with wearable device technologies for diagnosis and post-therapy of diseases.

## 1. Introduction

**Figure 1:**
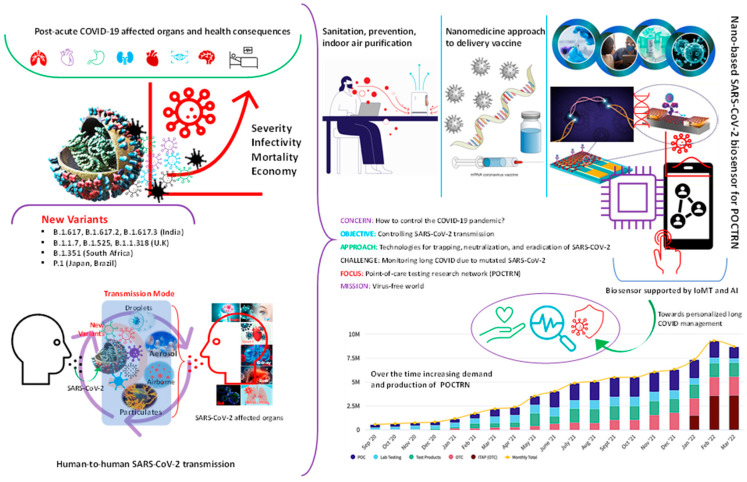
Schematic presentation of the status of the COVID-19 pandemic and recommendations on the development of smart sensors including wearable using a combinatory approach on biosensors supported with internet-of-medical-things (IoMT) and artificial intelligence (AI). The bar graph statistics on the bottom-right-hand side of the Figure are reprinted from https://www.nibib.nih.gov/covid-19/radx-tech-program/radx-tech-dashboard (accessed on 1 August 2022).

The COVID-19 virus had a devastating effect on human health throughout world. The world is slowly recovering from the COVID-19 menace. Initially, COVID-19 was a pulmonary disease, but it turned out to be a multisystem syndrome based on the evidence collected from the multitudes of patients [1,2,3,4,5,6]. The survivors of COVID-19 are experiencing multiple organ impairment (Figure 1). There are several reports about the health conditions of patients experiencing post COVID-19 complications terming them as long-term COVID effects [7,8,9,10]. The multisystem disease characteristic of post-COVID ailments is damaging vital human organs. Reports of complications in haematological system, cardiovascular system, gastrointestinal ailments, neurological disorder, lowering of immune system, brain haemorrhage, memory loss, etc., are being treated in several post-COVID patients [11,12]. Therefore, we require a comprehensive examination system for better control of the disease in personalized way [13,14].

In this situation, post-COVID-19 patients also need to be monitored continuously as long COVID have posed a bigger challenge for the governments to manage the human health as new ailments are being reported [8,15]. The post-COVID-19 patients are showing long-term COVID effects as efficiency of vital organs reduces leading to more complications [16,17,18,19,20,21,22]. Real time-polymerase chain reaction (RT-PCR) test for clinical diagnostics and computer tomography (CT-Scan) imaging of the lungs were the only two standard methods to detect and monitor COVID-19 infection [17]. However, the large-scale screening of population using above two methods for COVID-19 infection is expensive and time-consuming process for the government, as it cannot provide real time monitoring. Additionally, the RT-PCR test is conducted for screening and recurrence purposes, whereas we need more rigorous tests and investigations for monitoring of vital organs parameters. However, false positive tests and reliability of these tests pose a greater challenge to distinguish between healthy and infected people.

Moreover, mutations of COVID-19 virus are rising and causing multiple outbreaks in various countries [23]. COVID-19 has reached everywhere and contaminated our natural system including water, air, soil, etc., and continuously effecting human body in various ways. The long-term effect of COVID-19 infection is being reported in large numbers which needs to be controlled as post-covid patients have suffered permanent damages in various parts of the body (lung tissue damage, brain fog, severe mental illness, liver damage, impairment in eyesight, etc.) [8,24,25].

Therefore, COVID-19 and long COVID management require immediate attention from the government [26] (Figure 1). Regular diagnostic monitoring will be effective way for early detection, real-time monitoring of long COVID effects [27]. However, this is a huge task for the medical healthcare system to implement frequent monitoring for individuals due to lack of accessibility and limited available sources. Therefore, self-reporting of the symptoms and everyday monitoring could be a better way for long COVID management to track individual health parameters [28,29]. Hence, we urgently need portable, simple tracking systems to observe vital body parameters in regular intervals for long-sighted COVID management [30]. The common initial physiological signals for COVID-19 patients that are found to be an anomaly, such as respiration rate, variance in oxygen level, cough, heart rate variability, sleeping pattern, and raised body temperature [30]. Therefore, measuring or tracking the above-mentioned vital signs of human body can be designed to develop non-invasive predictive diagnostic technology for any disease detection including viral infections. These portable diagnostics tools including biosensors, microfluidic chips, digital patches, etc., can be used to track human vital parameters in real time [31,32,33,34,35,36].

Recently, wearable sensors are being explored for its simplicity, portability, and real time health monitoring system [37,38,39,40,41,42,43]. These smart devices can detect physiological changes in the human body providing a real time solution for quicker medical decision. Some of the well-known or prototype sensors have been instrumental for digital health monitoring, such as heart rate, O_2_ level, sleep pattern monitoring (smart watches), smart socks and shoes (motion sensor), temperature and stress mapping (smart textiles/tattoos), blood pressure (smart rings), throat infection (smart patches), breathing pattern and airborne pathogens (face mask), etc. [44,45]. Furthermore, Quer, G et al. developed a deterministic algorithm to discriminate between symptomatic individuals testing positive or negative for COVID-19 [46]. They have regularly analysed the changes in daily values of resting heart rate, length of sleep, and amount of activity, together with self-reported symptoms. Furthermore, passive and frequent monitoring of body parameters can be a practical solution for COVID-19, non-COVID-19, and post-COVID-19 patient using commercial wearables [47,48].

This present report provides a mini review highlighting the important features and facts about smart wearables that could be used for pre- and post-COVID-19 management. It also includes the current situation regarding the development and detection procedures being followed to combat this viral menace using wearable technology.

## 2. What Are Post-COVID Ailments?

Post-COVID conditions are a wide range of latest, complicated health issues that people are experiencing post-infection in various parts of the body [49]. Coronavirus infects the bronchial epithelial cells through (Angiotensin-converting enzyme 2) ACE2 receptors present in lung cells and spread infection to various body parts [50]. Patients who have recovered from COVID-19 infection still complaining about severe and chronic illnesses [51]. Short of breathing, chest pain, headache, diarrhoea, joint or muscle pain, fatigue, hormone imbalance, brain fog, etc. are some of the post-COVID-19 conditions are faced by the patients even months after infection [52].

There is no specific assessment to diagnose post-COVID-19 conditions. The symptoms of post-COVID-19 infection are very wide and diverse which make it more complicated to recognise or diagnose the disease condition [53]. Most of the time people do not realize that one is suffering from a post-COVID-19 condition, or that one is having any health issues as initial symptoms are not apparent enough. A post-COVID-19 infection has shown detrimental effect in different parts of the body, damaging lung functions, heart system, metabolic activity, nervous system, hormone release system, etc. [54] Breathing issues are most reported illness among the post-COVID-19 patients due to loss of activity of lung functions as virus impair lung tissues permanently. One of the major problems for long COVID infection is heart ailments including inflammation of the heart muscle, shortness of breath, rapid heartbeat, and shivering are some of the major symptoms of heart inflammation, Additionally, irregularity in blood flow system have been found after COVID-19 infection.

Another major issue that is persistent in long COVID patients are the mental health status [55,56]. There are plenty of reports on the phycological issues due to depression, anxiety, loss of life, financial burden, etc. Cerebral impairment post-infection is on the rise, which includes thinking, reasoning, and remembering things in daily life. Brain fog is one of the medical conditions where an individual become confused or disorganized, and unable to focus on small things. These leads to more complications to professional life and personal life which can be devastating. Another foremost issue with the long COVID patients is post-intensive care syndrome which occurs after prolong illness or hospitalization. Patient suffer multiple health issues including mental weakness. Post-traumatic stress disorder (PTSD) is another disorder that develops in some COVID-19 patients due to depression or anxiety.

Stress hormones can motivate you to perform well at workplace and meet your responsibilities. However, severe or long-term stress can affect your mental and physical health which can lead to severe anxiety and depression [57]. Many people get affected by stress post-COVID-19 ailment due to several reasons. Most of the patients are not able to sleep properly with worries going through their mind. Experiencing a major life change, such as the death of a loved one, an accident occurring, losing job, financial crisis, family issues, etc., can be detrimental to mental health. One may be at higher risk for stress-related problems if a person does not get enough sleep, which leads to poor health, including lack of emotional support. There are certain personalized approaches to reduce stress which can help your mental health condition and improve your quality of life. Some simple changes in regular lifestyle can improve the situation. Regular exercise, deep breathing, meditation, brisk walk, healthy diet, etc., can manage the stress levels.

Asthma is a long-term condition affecting children and adults post-COVID-19 ailments [58,59]. The most common symptom of asthma is coughing, short of breathing, wheezing, etc. The major cause of the asthma is small air passages in the lungs due to inflammation and tightening of the muscles around the small airways. COVID-19 related virus has worsened the condition of asthma affected person as lungs are affected severely. Asthma is not completely curable. However, good management strategy with inhaled medications can control the disease and asthma people can lead a healthy life.

## 3. Clinical Challenges Associated with Long COVID

Currently, there are several challenges for long COVID management as it is hard to predict when the COVID-19 outbreak will come to an end due to its mutation effect. Furthermore, the dearth of proper advice has caused frustration among the long COVID patients looking for better treatment. Self-medication, over the counter drug consumption, indiscriminate eating of multivitamins, dietary restrictions, etc., are also causing detrimental effect to body of several people. Inadequate support and lack of information from health care sector regarding health issues after COVID-19 has led to loss of confidence and dissatisfaction among the people towards government agencies.

It is often observed that COVID-19 recovered patients experience certain difficulties in daily activities. They complained about short of breathing, irregularity in bowel movement, loss of appetite, diabetes, sleep disorder, fatigue, muscle and joint pain, etc. [3] Apart from that, cured COVID-19 patients suffer from long ailments due to high doses of drugs intake. They require a personalized post-COVID-19 treatment on a regular basis and continuous monitoring of their vital organs. Therefore, it is very important to regularly keep an eye on the body parameters of the individual and record them on regular basis. It would be advisable for the individual to be alert about the deteriorating effects of the body activities and keep informing the physicians about health issues faced by the person. Therefore, it is high time to introduce wearables in our daily life for monitoring health parameters and provide real time medical solutions to the individuals.

Standard COVID-19 tests are invasive, time-consuming processes and are conducted when patients are already infected. The RT-PCR has served as the primary clinical laboratory diagnostic test for viral infection detection as these tests are reliable [60]. Furthermore, highly infected patients having severe chest infections go for secondary test, such as CT scan, to observe the lesions of pulmonary pneumonia in the lungs [61]. Even though these two methods were effective, their time-consuming procedure, delayed results, and limited availability create hindrances for mass screening.

**Figure 2:**
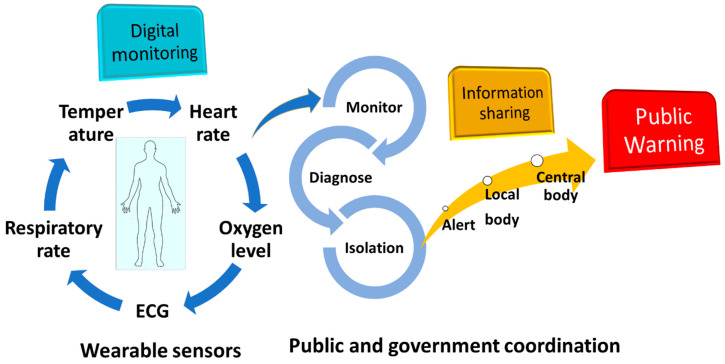
Role of wearable sensor technology for personal health monitoring in coordination with government for public healthcare management. ECG: electrocardiogram.

Additionally, there are large number of patients are not aware of the post-COVID-19 infection, lack of testing kits to screen the population, and some people are reluctant to report their symptoms to the government. Hence, they pose serious threat to the healthy population for another outbreak. Therefore, there is an urgent need for quick monitoring system that is easily accessible to the public for repeated measurement with high precision. With such a large population, wearable sensor technologies can be a viable approach to recognise post-COVID-19 ailments and emerging COVID-19 infection [37]. Figure 2 depicts a system for COVID-19 management as wearable sensors can monitor body vitals (body temperature, heart rate, oxygen level, respiratory rate, etc.) of individuals which can be analysed in real time for risk assessment for further diagnosis. If any person or certain population of a region found COVID-19 positive (or any chronic illness) based on the output of wearables, local authorities can issue a public warning. In this way, it can work as an efficient public warning system for any future outbreak of disease to contain the epidemic and can be used in emergency for chronic disease treatment.

## 4. Digital Biomarker and Wearable Sensors

The COVID-19 virus has caused a major health challenge globally due to the highly contagious nature of this virus [62,63,64]. The principal mechanisms by which the disease is transmitted are still being investigated. Thus, a quick diagnostic system is required to control this pandemic [65]. Biomarkers are characteristics of body that are quantitatively measured and analysed as an indicator of normal biologic processes, pathologic processes, or biological responses to a therapeutic intervention [66,67,68,69]. Currently, digital biomarkers are being explored for a non-invasive technique development for a larger population by means of digital devices such as portables, wearables, implantable, or digestible [44,66].

The digital biomarkers are the important factors to track the human body parameters for screening, monitoring of the post-COVID-19 affected patients [70]. Some of the relevant factors are oxygen level, body temperature, heart rate variability, ECG, sleeping pattern, coughing, etc. Fever and oxygen level measurements are the two most important parameters which can provide information towards differentiating healthy and unwell body conditions. Both the factors are equally important to predict and screen the other body parameters as high body temperature with lower oxygen level indicate premonition of any disease onset.

Fever is the most important clinical symptom of onset of any disease. However, continuous monitoring can give insights into the cause and nature of the disease for post-COVID-19 patients as it can relate to reinfection possibility or any other body ailments. The simplest mechanism of the body temperature measurement in wearables are temperature sensors which can continuously monitor skin temperature and alert any individual to take preventive steps.

The most significant parameter in determination of healthy human body is to track the oxygen level in circulatory system by measuring photoplethysmogram (PPG). PPG contains a light source and a photodetector which emits light to a tissue and the photodetector measures the reflected light from the tissue for measurement. Substantial drop in SpO_2_ level in human body (less than 95%) can cause brain damage, heart failure, or sudden death. So, tracking and monitoring of SpO_2_ level in wearables based on the light absorption characteristics of oxygenated, and deoxygenated haemoglobin in the blood oxygen saturation level. Most of the sensors are present in fingertips.

ECG is a medical diagnostic tool to evaluate the activity of the heart and provides the risk assessment of patient. Wearable watches and ECG patches can monitor the function of heart and provide a real time status of heart activity. These devices are consisting of a sensor system, a microelectronic circuit with a recorder and memory storage, and an internal embedded battery for recording and evaluating the heart rate of the patient.

Sleeping pattern measurement is another vital sign that reveals the psychological condition of the patient. Unusual sleeping pattern also reflects the low immunity. Hence, continuous, and remote monitoring of change in sleeping pattern in real time may help to prevent sudden events and reduce the possibility of severe condition.

Dry cough is one of the symptoms of disease. Therefore, monitoring of cough sound helps in the diagnosis and progression of the illness. Coughing signals are acquired with an audio or mechanical sensor that can detect the coughing sound through a microphone or a piezoelectric transducer. Cough can be identified automatically after the audio signal processing and pattern recognition through a set of algorithms.

**Figure 3:**
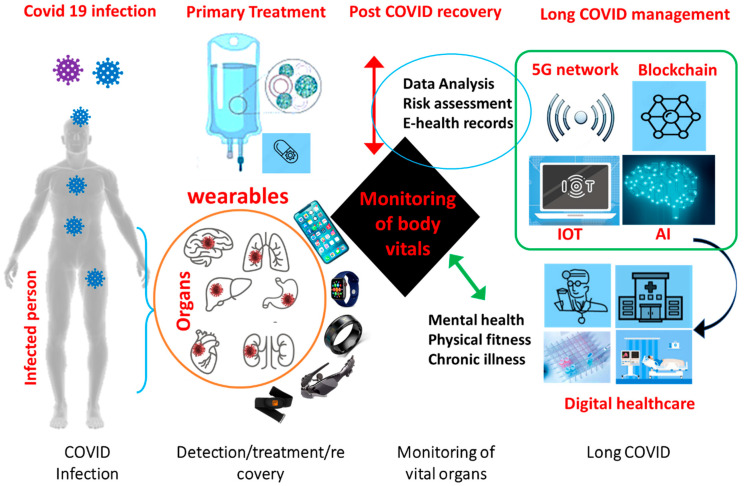
Application of smart wearable sensors for frequent monitoring of biometrics in synergy with IoMT and smart hospitals for post-COVID management.

The combination of wearable sensors and digital biomarkers have significant advantages over conventional sensors/devices, such as (i) non-invasive and remote access of the clinical data, (ii) immediate accessibility of digital data for clinical use, (iii) widespread availability of mobiles and smart watches, (iv) active and passive data collection, etc. [70,71]. Based on these data, AI and machine learning approaches can be utilized to predict the disease stage by correlating physiological metrics of daily living and human body performance of every individual [72]. The change in biometrics of human body recorded by wearables can be traced back by the health officials for disease analysis and proper steps can be taken to alleviate the spread of infection. A simple application of wearable sensors for long COVID management in combination with smart hospitals has been shown in Figure 3. Monitoring of body vitals of the infected person or post-recovery person for any organ damage can be screened and analysed based on the data provided by the wearables in real time, Additionally, IOT based system in conjunction with a 5G network can rapidly processed abundant of data (health parameters) using AI can help the doctors to take decision for further treatment [73].

## 5. How Wearable Sensor Can Manage Long COVID?

These wearable devices have an input for capturing body signals, such as optical sensor, camera, microphone, etc. For example, the apple watch is consisting of sensors which include an accelerometer and gyroscope for tracking of movement. At the back of watch, a ceramic based touch sensor which records heart rate with the help of photodiodes and LEDs. It measures pulse rate from the wrist using photoplethysmography (PPG) from an optical sensor which get analysed in real-time to evaluate pulse irregularity in human heart [74]. An innovative single chip known as S1 (system in package) powers the watch for conducting all the activities. The collected data is processed by proprietary algorithm developed by Apple for interpreting the signals and provide the digital data on screen.

Fitbit devices are electromechanical system equipped with a three-point accelerometer that count the number of steps depending on the distance travelled [75]. The accelerometer takes the movement data and translates it into digital measurements after differentiating various activities (walking, running, swimming, etc.). They also count the calories burned, oxygen level, and heart rate through an algorithm that captures the motion pattern of the individual.

Another interesting wearable is Oura ring which measures directly from finger arteries rather than the surface capillaries of wrist [76]. This enables Oura to capture the signal as it leaves the heart, rather than on a delay on the return. This ring uses a tool known as PPG or photoplethysmography in combination with infrared LED that measures heart rate, respiration, and heart rate variability through finger movement.

The WHOOP band has embedded sensors (LEDs, four photodiodes, and a body temperature sensor) that constantly monitor and track heart rate, heart rate variability, ambient temperature, accelerometery, and skin conductivity from wrist [77]. This metric is based on resting heart rate and max heart rate to calculate resultant cardiovascular exertion. Whoop records strain, recovery, and sleep based on the heart rate data. The higher heart rate, the more strain would be accumulated in the device. strain can be accumulated throughout the day based on the activity which records the heart rate.

The Biointellisense is a remote monitoring wearable device intended to collect physiological data in home and healthcare settings [78]. The data can include heart rate, respiratory rate, skin temperature, and other symptomatic or biometric data.

The detection of early COVID-19 and post-COVID-19 ailments is to investigate the body parameter changes in sensor data to symptom data which can be used to improve our ability to identify COVID-19 infection versus post-COVID-19 symptoms. Hirten et al. reported about a study on COVID-19 which found that changes in heart rate variability, captured by a smartwatch, signalled the presence of SARS-CoV-2 infection several days in advance of diagnosis [79].

Similarly, Quer at al. developed a smartphone app that collects smartwatch and activity tracker data, and self-reported symptoms and diagnostic testing results from individuals in the United States, and have assessed whether symptom and sensor data can differentiate COVID-19 positive versus negative cases in symptomatic individuals [46].

Similarly, there are group of researchers working towards smart system development using wearables and AI network which can help individuals to manage long COVID ailments. Wearables system can trace their symptoms to improve their quality of life for quantifiable data on how to do that. One such function is the Body Battery function on Garmin smartwatches uses physical activity, sleep, and stress levels to provide a composite score from 0–100 [80]. This score will let you know how much energy you have left to manage the symptoms and go for resting. For example, many people after COVID experiences fatigue, headaches, body pain, and lower heart rate on daily basis. These people have observed a common pattern that after a long hour of working, they experience a severity of their symptoms and, sometimes, the symptom get worse. Therefore, wearables can be used to analyse real time data to manage the body condition by choosing when to be active and when to rest.

Researchers using wearables to detect whether the body is in the early stages of fighting infection currently tend to focus on metrics related to heart rate, SpO_2_ level, step counts, skin temperature, etc. However, as wearables become increasingly capable of collecting precise data on body parameters, it would be easier to screen, monitor, and predict the disease condition for disease management. These wearables can be optimized with the help of AI to design working models for predicting, screening, and detection of the disease.

### Commercial Wearable Sensors

Fitness based wearable sensors has a promising market as people are more aware about their health and looking for simple and smart technology to keep an eye on their body vitals [81,82,83]. These highly sensitive tools can trace variations in the human body, such as heart rate variation, drop in oxygen level, increase in body temperature, etc. These irregularities in any individual could alert the system and raise an alarm for a possible clinical inspection immediately. In this way, these fitness sensors have the potential to become constant health monitoring system and could bring revolution as early detection system for many diseases, such as viral infection, water and air borne diseases, cancer, heart related diseases, etc. [84,85,86].

Some of the well-known wearable gadgets that are available in the market are Fitbit, Apple watch, Oura, Empatica, Whoop, Garmin, Biointellisense, Biobeat, etc. [87,88]. Most of them are capable to measure vital body parameters, such as heart rate (HR), heart rate variability (HRV), blood oxygen saturation (SpO_2_), resting heart rate (RHR), and respiration rate (RR) [89,90,91,92]. Additionally, Biointellisense, Oura, and Biobeat can provide skin temperature (ST) [93]. The irregularity in physiological signals are the early symptoms of any diseases and could be used as a potential early detection platform for COVID-19 infection. The measurement of these vital body parameters are part of a wearable health-monitoring system and their real-time information about body signals can provide in situ health condition. Among the above-mentioned wearable sensors, Apple, Bioitellisense, and Biobeat have been approved by FDA and are currently under trial for development of early warning system for COVID-19 infection [37]. Table 1 shows the some of the well-known commercial wearables being used in market for personal healthcare monitoring.

Scripps Research Institute in collaboration with Apple Watch is working on heart measurements to find a possible way of tracking the coronavirus infection [37]. The Apple watch provides monitoring of ECG used to assess the activity of the heart and provide the risk assessment of patient. They are particularly interested whether heart rate measurement is enough for clinical symptoms of any viral infection. Another company Whoop has developed an armband/wristband for monitoring heartrate, oxygen level, skin temperature, respiration rate, heart rate variability, etc., by subscribing with them for health management.

Similarly, Stanford University, Scripps Research Institute and Fitbit running a joint investigation for a possible early warning system development based on algorithm to predict the inception of COVID-19 viral infection by measuring the heart rate, oxygen level saturation, and quality of sleep of a person [94]. Fitbit company claimed that their devices were able to predict nearly half of COVID-19 cases even before they developed any symptoms of coronavirus infection. According to Professor Ryan Shaw of Duke University, the current pandemic of coronavirus is very significant and unique for clinical study as it provides a large amount of data regarding an infection. He is leading a team of researchers for developing a tool known as ‘Covidentify’ by monitoring sleeping pattern, heart rate, and oxygen level of a person for Coronavirus infection detection [37].

Another promising wearable tool is Oura ring which can monitor physiological parameters of COVID-19 infection [73]. Researchers at West Virginia University of Rockefeller Neuroscience Institute has developed an app combining the Oura ring to predict the symptoms of Coronavirus infection by monitoring surface temperature of body, heart rate, respiration rate, etc. The research team reported that their sensor is 90 percent accurate at predicting early warning signs for coronavirus. Their research is based on data collected from 600 health-care workers.

Empatica in collaboration Biomedical Advanced Research and Development Authority (BARDA) has developed an COVID-19 early detection system named ‘Aura’ using their wearable technology [38]. This non-invasive smartwatch technology provides a real-time information about COVID-19 infection before symptoms appear by measuring respiratory rate, heart rate, and peripheral temperature of the concerned person. The smartwatch Aura can send an early warning to the consumer about his/her deteriorating health conditions.

At Florida International University (FIU), Prof. Shekhar Bhansali and his team are working on prototype wearable sensor fabrication. His team has constructed an ‘electronic nose’ which can sense alcohol odour from the skin in a fraction of second and measure blood alcohol level to address alcohol abuse [94]. This wearable sensor can also be used as digital thermometer to measure surface temperature from skin surface for development of a possible virus infection detection. The Human cyber-physical systems (HCPS) laboratory in FIU are developing smart sensors for behavioural and physiological monitoring and analysis of human body. Some of the wearable devices are high-fidelity wearable ECG (electrocardiogram) and wearable EEG (electroencephalogram) to detect heart and brain activity for understanding sleep pattern, stress testing, etc. [95] Similarly, another group in FIU have developed a portable imager as a low-cost alternative to MRI/CT devices for brain implants for continuous real-time tissue characterization using wearable sensors. Shan et al. developed a nanomaterial-based sensor array with multiplexed capabilities for detection and monitoring of COVID-19 from exhaled breath [96]. The fabricated sensors are composed of different gold nanoparticles linked to organic ligands, creating a diverse sensing layer that can swell or shrink upon exposure to volatile organic compounds (VOCs), causing changes in the electric resistance.

## 6. Discussion

There are quite a few diagnostic tests (i.e., molecular tests and ‘rapid’ antigen tests), serology, or antibody tests are available in the market for any infectious disease management. The rapid antigen test is sensitive enough to screen the large population using a nasal or throat swab, or from saliva by spitting into a tube and quick results can be expected depending on the presence of large health workers for quick conduction and evaluation of test. Another sensitive way of detecting the COVID-19 infection is to conduct antibody tests that measure the presence of antibodies of COVID virus from serum, plasma, or whole blood collected from human body, Additionally, LFA were introduced for large scale screening of populations as viral antigen test kit to control outbreak by tracing the infected persons. This kit is very useful to produce a rapid result, are inexpensive and easy to operate. Currently, the gold standard for detection of COVID-19 infection is reverse RT-PCR. All these tests have a turnaround time of 2–3 days. All of the technologies have the advantage for screening and cheaper option for one-time screening and detection. However, for multiple screening and continuous monitoring of the population, these systems are not feasible. Plenty of health workers, millions of test kits along with infrastructure facilities would be required which will incur a huge expenditure for the government agencies.

On the other hand, wearables have the potential to minimize the cost of screening and monitoring of the pre- and post-COVID management. These devices can be used for scaling up of mass testing to aid track and trace methodology. Furthermore, these sensitive devices can alert the authorities to take immediate action after the risk assessment of the huge data provided by the wearables in real time. With the help of AI and the IoMT enabled system, faster communication will help to take quick decisions for any emergency. There is additional benefit of providing the results in a much shorter time frame.

There is no comprehensive study regarding the long COVID management using wearables. Most studies have been conducted on COVID-19 management using wearables and their studies have been analysed for pre-infection prediction, current COVID-19 symptoms, and post-COVID-19 complications. Increased heart rate was most frequently associated with COVID-19 infection, change in sleeping pattern along with increased skin temperature, and respiratory rate were recorded in most cases. One of the earliest studies was conducted by Mishra et al. [84]. They investigated physiological and activity data from 32 individuals infected with COVID-19, identified from a cohort of nearly 5300 participants, and found that 26 of them (81%) had alterations in their heart rate, number of daily steps, or time asleep. They also suggested that activity tracking and health monitoring via consumer wearable devices may be used for the large-scale, real-time detection of respiratory infections, often pre-symptomatically. According to Scripps research digital trials centre for long COVID wearable study, out of all people diagnosed with COVID-19, it is estimated that 10–30% have long COVID, including 17 million in the United States and 75 million worldwide.

## 7. Challenge of Connecting Wearable Sensors for Long COVID Management

The future scope of wearable sensor is huge as it can transform the digital health technology [97,98]. The introduction of digital health in individual life is very significant step toward developing robust platforms for pre and post-COVID-19 management of patients. However, there are few challenges for proper implementation of these devices. Some of the factors that concern many individuals are data privacy, human ethics, legality, an unwanted burden of digital monitoring, etc. [71]. The public use of any individual clinical data is a big concern. There is high probability of manipulating or misusing of the data usage especially for tools that collect near-continuous data, such as movement (location), vocal sound, and other sensitive biometric states [66]. However, some company for example: WHOOP has a fair data policy and seek permission from individual participants about sharing their data which will be used for research purposes only [99]. This kind of fair data use agreement for handling such sensitive issue would be ideal for commercial device development.

It is not feasible to wear multiple devices at one time, but it can be chosen wisely based on the requirement of the individual. For example, to monitor the oxygen level and heart rate simultaneously, one can wear smart watch or heart-based wearable providing data for these two health parameters. Similarly, smart facemask can provide real time data of the breathing pattern and can also provide information for any mouth/throat related infection [100]. Therefore, it would be highly beneficial for personalized nanomedicine development for quick diagnosis and long-term investigation of chronic diseases. This type of research would lead to innovation in health management and would provide us a salubrious lifestyle.

Wearable devices require a higher level of digital knowledge, though automatic functions can alert the users. Wearables can be highly effective for older people, who are considered less skilled with technology. Moreover, these wearables are also very expensive for most people to afford. Therefore, it would take few more years to adopt this technology for everyone as simpler and cheaper wearables will be available in future.

Furthermore, digital data collected by the device are interpreted by a proprietary algorithm of that company which is subject of concern for patient and doctors over its credibility. This translational research which are based on algorithm raises genuine apprehension among the patients about its sensitivity in clinical management. It is too early to decide any pros and cons of this new technology, but joint effort by the industry, clinicians, civilians, and government would be required to conquer over this pandemic. However, these innovative technologies can also be used to design an early warning system to stop epidemic of disease, rare disease tracking, and personalized medicine development for a patient suffering from a chronic disease, which requires continuous monitoring and frequent diagnosis.

## 8. Conclusions and Viewpoint

The future of wearable technology is to introduce an implantable medical device into the human body through surgery. Current existing implantable medical devices are pacemakers, stents, cochlear implants, etc., which are used for conducting various organ functions. This prototype wearables will deliver medication such as pain relief, organ monitoring, and regulation of body functions, such as heart rate, blood sugar level, metabolic rate, etc. They will provide support to organs, rebuilding body functions, and achieving a better quality of life. This futuristic implantable system will work continuously for remote monitoring of various body ailments, such as asthma, brain fog, depression, hormonal imbalance, eyesight, physical activity, etc. These devices can collect clinical symptoms, such as coughs, body temperature, heart rhythm, oxygen level, metabolic rate, etc., from patients providing real-time data and promoting self-management for chronic conditions. Currently, deep brain neurostimulators, gastric stimulators, cardiac defibrillators, insulin pumps, etc., are being investigated for clinical testing for long-term health improvement. All these implants can be monitored through digital platform (AI and ML based IoMT network) for real-time analysis of health [14,101].

The introduction of 5G and AI in the field of healthcare management has shown improvement for digital healthcare system. The presence of healthcare professionals and hospitals around the country with sophisticated instruments are limited. Researchers are exploring 5G network-based system for health management. A personalized emotion-aware healthcare system was developed by researchers using 5G that emphasizes on the emotional care, especially for children, and mentally ill and elderly people [102].

**Figure 4:**
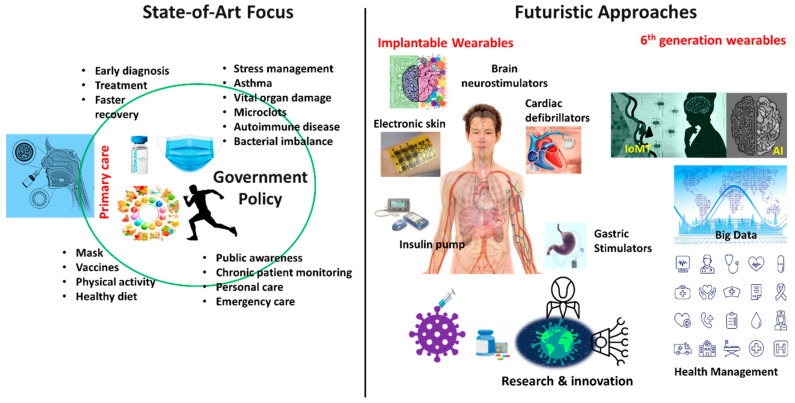
Depicting the future approach for management of long COVID through implantable wearables based on digital health care (IoMT and AI based cloud computing), and show a government focus on awareness, research, personal medicine, etc.

Several AI based IoMT system are being explored for digital healthcare system and achieved remarkable progress in screening, monitoring, disease diagnosis, and predicting the disease outcome [26]. However, one of the biggest challenges is model training, building internet-based network for deployment of IoMT devices with large memory sources. We require more real-time monitoring analysis system for investigation of vital parameters for regular clinical assessment of the patients. More work is needed in this direction to expand and involve active participation of individuals and government agencies for a successful infrastructure development to reduce the channel congestion and smooth communication of abundant data movement. The recent focus and future approaches of connecting wearable technology to manage long COVID for a successful health management is illustrated in Figure 4.

The market of electronic consultation (E-consultation) has increased over the years. This is due to lack of transport facilities, unavailability of doctors, remote presence of patients, and the unwillingness of senior citizens to go to hospitals. There are various mobile applications are coming up (such as Practo mobile app) for patients to get connected to their doctors for 24 h consultations for initial screening, follow-ups, and second opinions. Many hospitals and prominent doctors have availed this technology for their outreach to existing and new patients.

There is no specific treatment available for the post-COVID-19 patients. The treatment strategies vary according to patient symptoms and conditions. However, based on preclinical evidence and previous experience of SARS cases some of the well-known techniques/drugs or combination of both have been found to be effective for some section of the critical patients. Even though vaccines have managed to control the epidemic and lower the mortality rates, we still need systematic long COVID management system. Wearable technology produces myriad datasets from individuals and set of populations which can be combined with computational systems (AI and machine learning system) to predict the future.

Researchers have investigated the accuracy of a machine learning model for the detection of post-COVID-19 infections/complications collected from various wearable sensors and provided well defined model for better management of the individuals for diagnosis, treatment and need for hospitalization. Moreover, some of the well implemented measures were taken that have reduced mortality rate due to rapid identification, prevention, and control, followed by patient isolation, diagnosis, and contact tracing to control further transmission among population. The development of new technologies, such as AI, smart sensors, point of care tools, etc., have opened new opportunities to combat this deadly virus and have shown promising results so far [103]. Developing a complete infrastructure of digital health platforms for monitoring and managing any disease in conjunction with government, local bodies, and the individual will bring significant change for the healthcare system (Figure 4).

## Figures and Tables

**Table 1:** Some of the commercial wearables for biometric measurements. ECG: electrocardiogram, O_2_: oxygen.

Wearable	Type	O_2_ Level	Heart Rate	Respiratory Rate	Temperature	Other
Apple watch	Wrist	yes	Yes	yes	no	ECG
Fitbit	Wrist	yes	Yes	yes	yes	Sleep
Oura	Ring	no	Yes	yes	yes	Sleep
Hexoskin	Shirt	yes	Yes	yes	no	Sleep
Whoop	Arm/wrist	no	Yes	yes	yes	Sleep
BioIntelliSense	Patch	no	Yes	yes	yes	Sleep, coughing
Garmin	Wrist	yes	Yes	yes	no	sleep
Biobeat	Wrist/patch	yes	Yes	yes	yes	Blood pressure, ECG

## Data Availability

Not applicable.
